# Method to grow *Actinobacillus pleuropneumoniae* biofilm on a biotic surface

**DOI:** 10.1186/1746-6148-9-213

**Published:** 2013-10-20

**Authors:** Yannick DN Tremblay, Cynthia Lévesque, Ruud PAM Segers, Mario Jacques

**Affiliations:** 1 Groupe de recherche sur les maladies infectieuses du porc, Faculté de médecine vétérinaire, Université de Montréal, 3200 rue Sicotte, St-Hyacinthe, Québec J2S 7C6, Canada; 2 Microbiological R&D, MSD Animal Health, 5831 AN, Boxmeer, The Netherlands

**Keywords:** Biofilm, Biotic surface, SJPL cell line, *Actinobacillus pleuropneumoniae*, Confocal laser scanning microscopy, Host-pathogen interaction

## Abstract

**Background:**

*Actinobacillus pleuropneumoniae* is a Gram-negative bacterium and a member of the *Pasteurellaceae* family. This bacterium is the causative agent of porcine pleuropneumonia, which is a highly contagious respiratory disease causing important economical losses to the worldwide pig industry. It has been shown that *A. pleuropneumoniae* can form biofilms on abiotic surfaces (plastic and glass). Although *in vitro* models are extremely useful to gain information on biofilm formation, these models may not be representative of the conditions found at the mucosal surface of the host, which is the natural niche of *A. pleuropneumoniae*.

**Results:**

In this paper, we describe a method to grow *A. pleuropneumoniae* biofilms on the SJPL cell line, which represents a biotic surface. A non-hemolytic, non-cytotoxic mutant of *A. pleuropneumoniae* was used in our assays and this allowed the SJPL cell monolayers to be exposed to *A. pleuropneumoniae* for longer periods. This resulted in the formation of biofilms on the cell monolayer after incubations of 24 and 48 h. The biofilms can be stained with fluorescent probes, such as a lectin against the polymer of N-acetyl-D-glucosamine present in the biofilm matrix, and easily observed by confocal laser scanning microscopy.

**Conclusions:**

This is the first protocol that describes the formation of an *A. pleuropneumoniae* biofilm on a biotic surface. The advantage of this protocol is that it can be used to study biofilm formation in a context of host-pathogen interactions. The protocol could also be adapted to evaluate biofilm inhibitors or the efficacy of antibiotics in the presence of biofilms.

## Background

*Actinobacillus pleuropneumoniae*, a Gram-negative bacterium belonging to the *Pasteurellaceae* family, is the causative agent of porcine pleuropneumonia. This severe and highly contagious infectious respiratory disease causes major economic losses in the swine industry [1,2]. *A. pleuropneumoniae* is transmitted by means of aerosols or by direct contact with infected animals. The infection may result in rapid death or in severe pathology [1]. Animals exposed to *A. pleuropneumoniae* may develop chronic infections or become asymptomatic carriers and these animals may become the source of transmission of the disease to healthy animals or herds [1]. The virulence factors involved in colonization and lung lesions, which include type IV fimbriae, lipopolysaccharides, and the pore forming RTX toxins ApxI to IV, have been well characterized (for a recent review see [2]). The role of biofilm formation in the pathogenicity of *A. pleuropneumoniae* is gaining recognition.

Bacterial biofilms are structured clusters of bacterial cells enclosed in a self-produced polymer matrix that are attached to a surface [3-5]. Bacteria can adhere to a biotic surface (e.g. cells at the mucous layer) as well as to abiotic surfaces (e.g. floor or equipment found at a farm). The polymer matrix is often composed of exopolysaccharides, proteins and nucleic acids. The biofilm protects bacteria from hostile environmental conditions. Bacteria within a biofilm can resist attack from the host immune response, and are less sensitive than planktonic cells to desiccation and to the action of biocides.

It has been clearly shown that *A. pleuropneumoniae* has the ability to form biofilms under static growth conditions in polystyrene microtiter plates [6,7] or with agitation in glass tubes [6,8]. Using a 96-well microtiter plate assay, we screened mutants obtained by transposon mutagenesis for their biofilm phenotype and identified unique genetic determinants associated with biofilm formation in *A. pleuropneumoniae*[8]. We also demonstrated that the biofilms of *A. pleuropneumoniae* field isolates were more resistant than their planktonic counterpart to ampicillin, florfenicol, tiamulin and tilmicosin [9]. Recently, our group demonstrated that *A. pleuropneumoniae* can form a biofilm on a glass slide under low-shear force in a drip-flow apparatus [10]. The biofilm formation on abiotic surfaces (plastic and glass) depends on the production of a polymer of β-1,6-N-acetyl-D-glucosamine (PGA) [10-12]. Although *in vitro* models used in biofilm studies are extremely valuable to acquire knowledge regarding biofilm formation, the main limit of these *in vitro* models is that they do not mimic exactly the conditions found in the host such as the lung mucosa, which is a natural niche for *A. pleuropneumoniae*. Therefore, the aim of this study was to develop a method to form biofilms on a biotic surface. We selected the SJPL cell line because this cell line has been used extensively in our laboratory to study adherence of *A. pleuropneumoniae* and other porcine bacterial pathogens to cells [13]. In addition, this cell line was found to be permissive to porcine reproductive and respiratory syndrome virus (PRRSV) infection [14] and therefore represents a potential and powerful model to study viral-bacterial co-infections.

## Results and discussion

**Figure 1:**
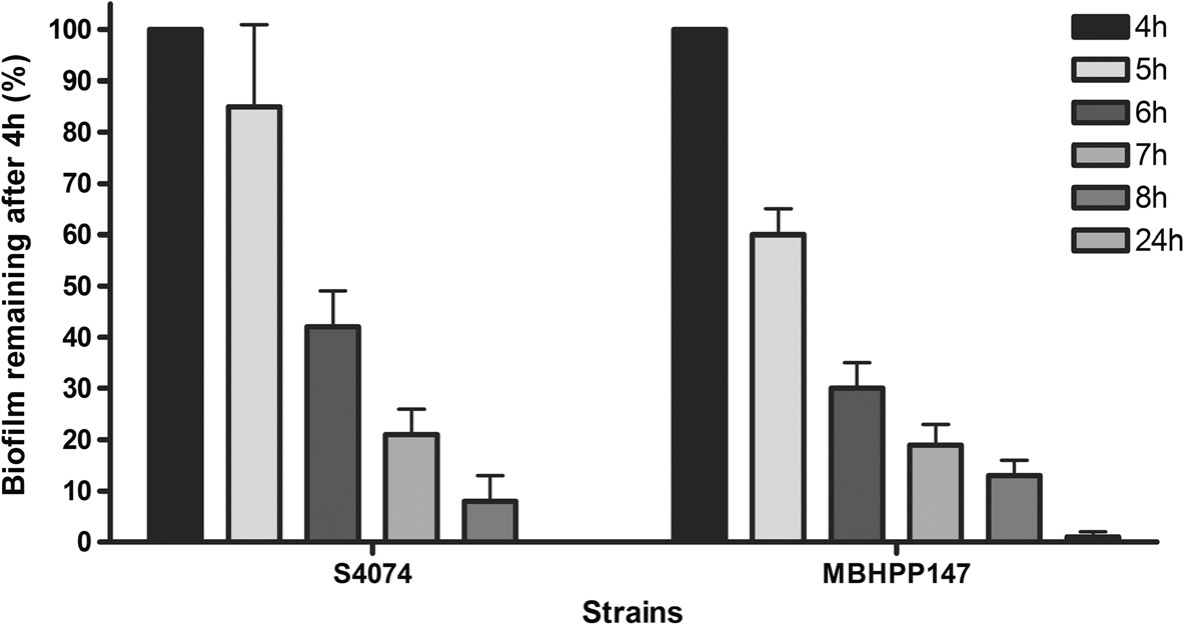
**Effect of incubation time on the biofilm dispersion of *A. pleuropneumiae *** **strain S4074 and MBHPP147 in 96-well polystyrene microtiter plates.**

**Figure 2:**
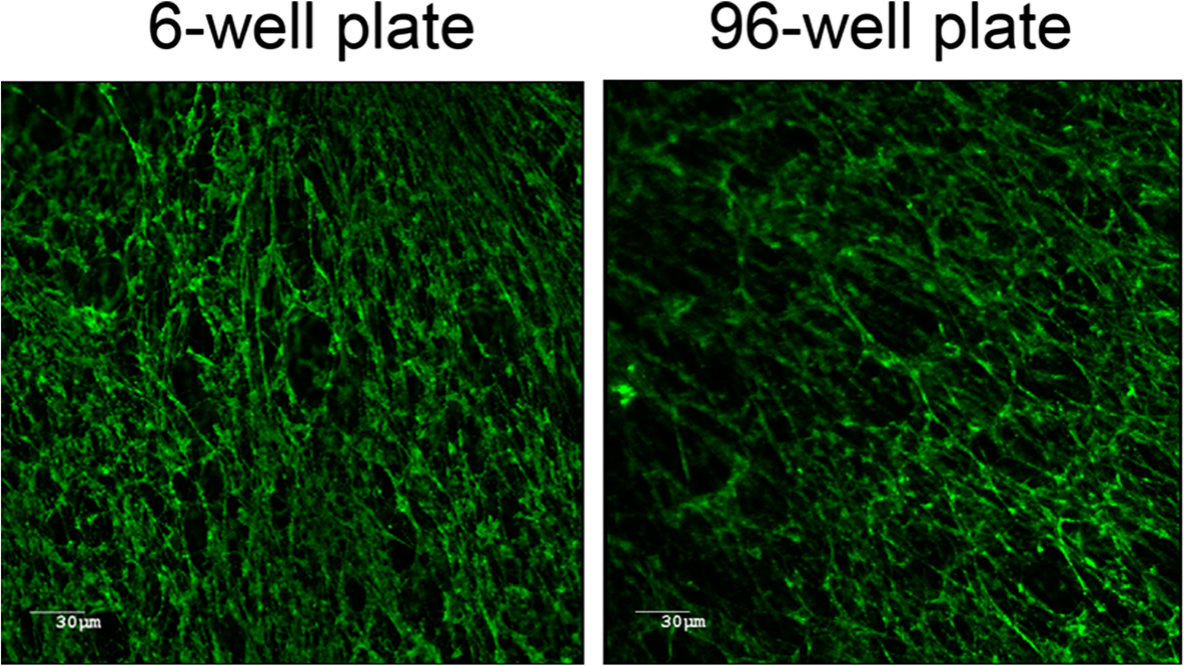
**Confocal laser scanning microscopy images of 6-hour biofilms of *A. pleuropneumoniae *strain MBHPP147 stained with WGA-Oregon green 488.** Biofilms were formed in 6-well and 96-well polystyrene plates.

A non-hemolytic strain of *A. pleuropneumoniae* with deletions in the *apxIC* and *apxIIC* genes, named MBHPP147, was used in this study. We first determined the ability of this strain to form a biofilm using a standard microtiter plate assay combined with crystal violet staining (Figure 1). Strain MBHPP147 was able to form biofilms on the plastic surface and, as previously observed with the *A. pleuropneumoniae* S4074 parent strain [7], a robust biofilm was formed within 4 hours but rapidly dispersed afterwards. Such rapid dispersion has been observed previously with strain S4074 but the exact cause has yet to be identified [10]. The presence of the biofilm was confirmed by confocal laser scanning microscopy by staining the biofilm matrix with Wheat Germ Agglutinin (WGA)-Oregon green 488 (Figure 2). This lectin is known to bind to the PGA present in the biofilm matrix of *A. pleuropneumoniae*[11,12].

We then verified that strain MBHPP147 was also non-cytotoxic for the SJPL cells. Cytotoxicity was measured using the level of activity of lactate dehydrogenase (LDH) released from cells. Cells were incubated for up to 96 hours without or with bacteria and no statistically significant differences were observed between the % cytotoxicity of the uninfected controls and the SJPL cells incubated with strain MBHPP147 (Table 1). For strain S4074, cytotoxicity reaches over 50% after 3 hr of incubation with SJPL cells (data not shown). Therefore strain MBHPP147 is a suitable *A. pleuropneumoniae* mutant for protocols, such as biofilm formation, requiring long incubation periods with animal cells.

**Table 1:** **Cytotoxicity as measured by lactate dehydrogenase (LDH) released by SJPL cells incubated with *A. pleuropneumoniae *** **strain MBHPP147 for a period of up to 96 hours**

**Incubation time (h)**	Cytotoxicity (%)*	
	**Control SJPL cells**	**SJPL cells infected with *A. pleuropneumoniae***
**24**	0	0
**48**	4.93 ± 4.93	14.67 ± 7.55
**72**	10.60 ± 10.60	8.15 ± 8.15
**96**	21.05 ± 21.05	22.65 ± 20.65

*The % represents the released LDH/ total cellular LDH and the data are the means ± SEM of three independent experiments with duplicates. No statistical differences were observed between the control cells and the cells infected with *A. pleuropneumoniae* strain MBHPP147 (two-way ANOVA).

**Figure 3:**
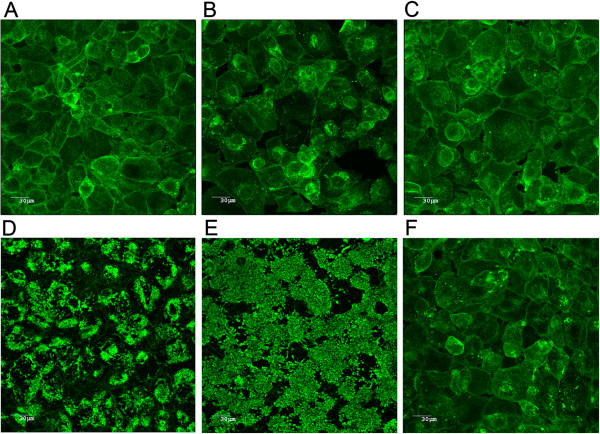
**Confocal laser scanning microscopy images of biofilms of *A. pleuropneumoniae *strain MBHPP147 grown on SJPL cells and stained with WGA-Oregon green 488.** Uninfected SJPL cell control (48 hours post mock infection) **(A)**; SJPL cells incubated with *A. pleuropneumoniae* strain MBHPP147 for 3 hours **(B)**, 6 hours **(C)**, 24 hours **(D)**, 48 hours **(E)** or 48 hours and treated with Dispersin B **(F)**.

**Figure 4:**
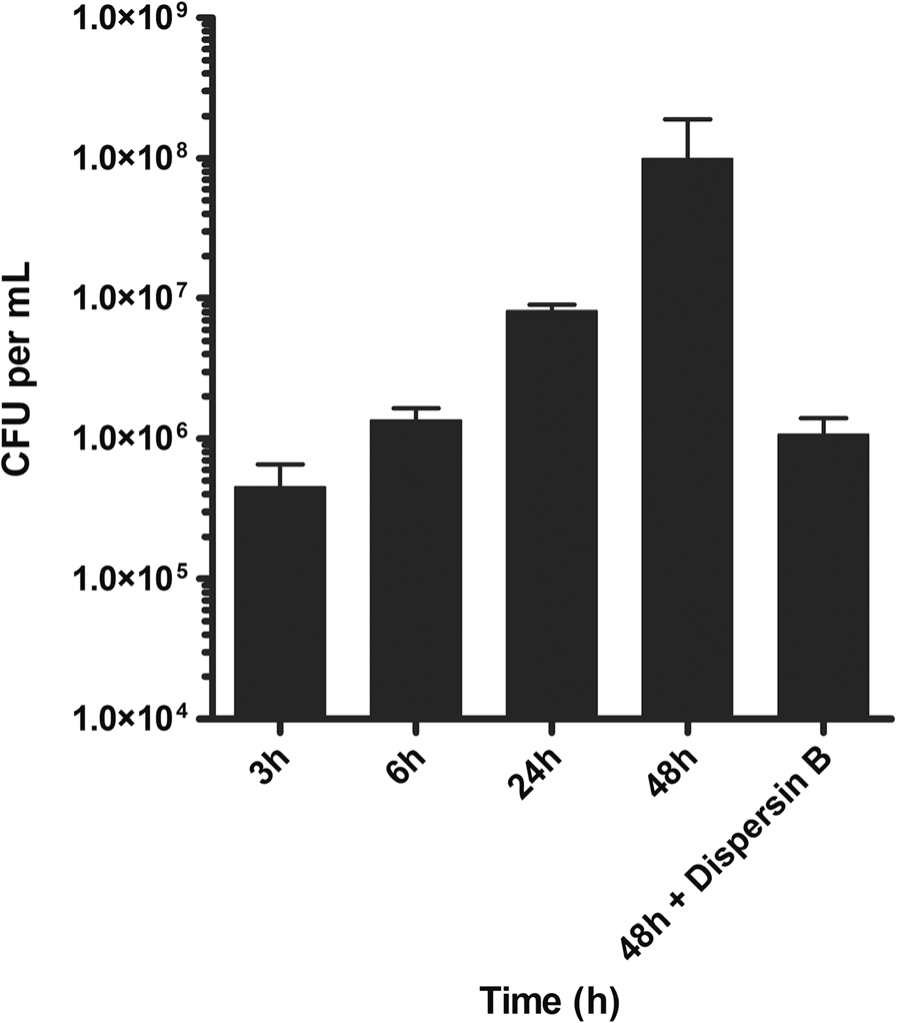
**Number of colony forming units (CFU) attached to SJPL cells. SJPL cells were infected with *A. pleuropneumoniae *** **strain MBHPP147 and attached cells were recovered after 3, 6, 24, and 48 hours of incubation and after a Dispersin B treatment (48 h + Dispersin B).**

**Figure 5:**
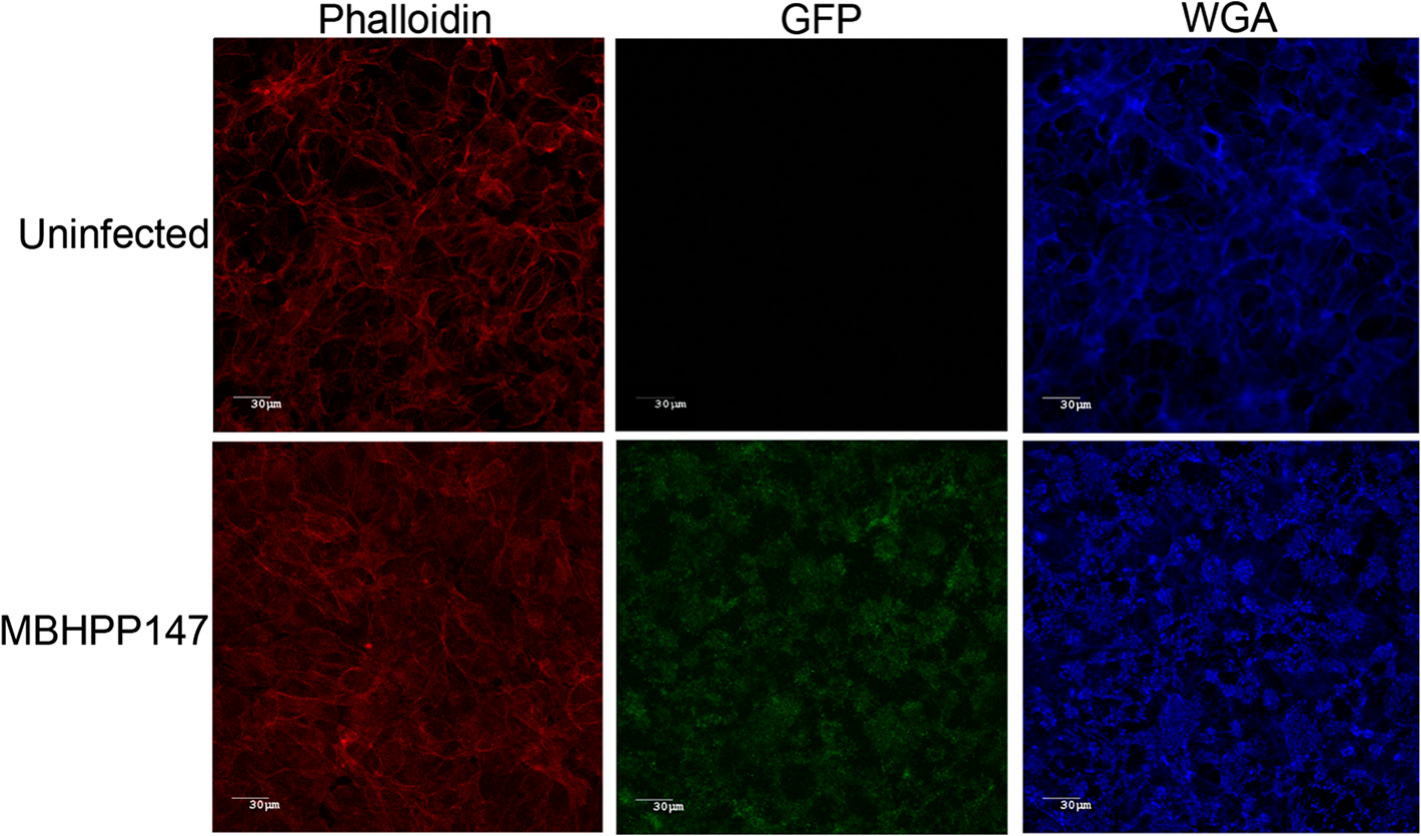
**Confocal laser scanning microscopy images of a 24 hour biofilm of *A. pleuropneumoniae *** **strain MBHPP147 expressing GFP grown on SJPL cells and stained with Phalloidin-AlexaFluor 594 and WGA-AlexaFluor 633.**

The next step was to evaluate the ability of strain *A. pleuropneumoniae* MBHPP147 to form biofilm on a biotic surface (i.e. on a SJPL cell monolayer). Therefore, *A. pleuropneumoniae* MBHPP147 was incubated with SJPL cells for up to 48 hours. Confocal laser scanning microscopy and staining with WGA-Oregon green 488 was used to visualize the biofilms (Figure 3). Uninfected SJPL cells were used as a negative control, and, as expected, were devoid of any adherent bacteria (Figure 3A). The cells were stained with WGA because this lectin binds to N-acetyl-glucosamine and N-acetyl-neuraminic residues. There was no obvious biofilm formation after 3 h (Figure 3B) and 6 h (Figure 3C) of incubation despite the attachment of *A. pleuropneumoniae* MBHPP147 on SJPL cells (Figure 4). *A. pleuropneumoniae* MBHPP147 clearly formed robust biofilms at the surface of SJPL cells after 24 hours (Figure 3D) and 48 hours (Figure 3E) of incubation. The formation of biofilms was associated with an increased in the number of bacteria attached to SJPL cells (Figure 4). The 24 h and 48 h biofilms were stained with WGA suggesting the presence of PGA in the matrix as observed previously with abiotic surfaces [7,10-12]. The presence of PGA was confirmed by the dispersion of the biofilm (Figure 3F), and the decrease in the number of bacteria attached to SJPL cells (Figure 4) when the biofilms were treated with Dispersin B, a glycoside hydrolase that catalyzes the hydrolysis of poly-N-acetylglucosamine. To further ensure that the WGA staining observed was associated with the biofilm of *A. pleuropneumoniae* MBHPP147, the assay was repeated with a bacterial strain expressing a plasmid encoded GFP from a constitutive promoter [15]. A GFP signal was detected for SJPL cells infected with *A. pleuropneumoniae* and it was not detected when cells were not infected (Figure 5). This indicated that bacteria were attached to the cells. Furthermore, the GFP pattern exhibited by the bacteria on SJPL cells was very similar to the one observed with WGA staining (Figure 5). Additionally, the WGA staining observed in the uninfected cells appeared to follow the pattern of the phalloidin staining of the cytoskeleton (Figure 5). Taken together, this indicated that the observed change in WGA-staining pattern (Figures 3 and 5) is clearly due to the formation of biofilm by *A. pleuropneumoniae* and not due to a structural change on the surface of SJPL cells*.*

Unlike biofilms formed on an abiotic surface [10], the biofilms formed on SJPL cells was present after 24 and 48 hours. Furthermore, *A. pleuropneumoniae* S4074 and MBHPP147 were not able to form biofilms in the culture medium used to grow SJPL cells in the absence of SJPL cells (data not shown). Taken together, these observations underline the importance of bacterium-animal cell interactions in biofilm formation and the use of a biotic surface. This conclusion is also supported by the fact that exposing *Streptococcus pneumoniae* to the airway epithelial cells 1HAEo-before a biofilm assay resulted in a significant increase in biofilm formation in microtiter plates [16].

Only a few co-culture models have been described in the literature to study human respiratory tract pathogens. For example, *S*. *pneumoniae* can form biofilms on NCI-H292 (mucoepidermoid bronchial carcinoma cells) and primary human bronchial epithelial cells [17]. While the majority of primary bronchial epithelial cells remained intact after 24 hours of incubation with *S. pneumoniae*, the NCI-H292 cells were rapidly killed by the bacteria. Recently, Vidal et al. [18] developed a biofilm reactor with living cultures of human-derived lung cells A549 and a continuous flow of nutrients to avoid the cytotoxic effect of *S. pneumoniae*. Another example is *Pseudomonas aeruginosa* that can form biofilms on cystic fibrosis-derived human airway epithelial cells [19]. However, the biofilm disrupted the monolayer after an overnight incubation in the absence of antibiotics. To the best of our knowledge, this is the first report of biofilm formation by *A. pleuropneumoniae* on a biotic surface and for long incubation periods. In previous works [13], we have determined the SJPL cells’protein profile after a short incubation of 3 hours with *A. pleuropneumoniae* as well as the transcriptomic profile of *A. pleuropneumoniae* during that interaction. It will be interesting in future studies to use this new protocol to investigate the SJPL cell’s response to bacterial biofilms present after 24 and 48 hours of co-culture. It will also be interesting to compare *A. pleuropneumoniae* gene expression in a mature biofilm formed on a biotic surface with the genes expression determined with mature biofilms on plastic (polystyrene microtiter plate) or glass (drip-flow apparatus) surfaces [10]. Additionally, the protocol could be adapted and design to screen for anti-adherence and/or anti-biofilm molecules.

## Conclusions

The use of a non-hemolytic, non-cytotoxic strain of *A. pleuropneumoniae* allows for longer incubation periods with an *in vitro* cell line model. Others have used paraformaldehyde-fixed epithelial cells to avoid the cytotoxic effect of a bacterial pathogen [17]. Although cell monolayers do not perfectly reproduce the *in vivo* conditions, the use of a biotic surface is certainly more physiologically relevant to study biofilm formation than a plastic or a glass surface. This model could be easily adapted to evaluate inhibitors of bacterial adherence or inhibitors of biofilm formation, or the efficacy of antibiotics in the presence of a biofilm. Finally, this model can also be adapted to study bacterial-viral co-infections knowing that the SJPL cells are permissive to PRRSV [14].

## Methods

### Bacterial strain

*A. pleuropneumoniae* MBHPP147 is a non-hemolytic derivative of the serotype 1 reference strain S4074. Construction of strain MBHPP147 is described in patent EP 0810283A2. Briefly, a streptomycin and nalixidic acid resistant mutant of *A. pleuropneumoniae* strain 4074 (MBHPP104) was mated with an *Escherichia coli* SM10 λpir strain carrying a plasmid containing a deleted *apxIC* gene. Positive exconjugants were selected on solid medium containing nalixidic acid and gentamycin and a mutant with reduced haemolytic activity on solid CBM containing 2% sheep erythrocyte was selected and named MBHPP111. Once the deletion was confirmed, this mutant was then mated with an *E. coli* S17-1 λpir strain carrying a plasmid containing an in-frame deletion in the *apxIIC* gene. Exconjugants were selected on solid media supplemented with nalixidic acid and gentamycin. A mutant lacking hemolytic activity on blood agar was selected and was named MBHPP147. In summary, strain MBHPP147 contains deletions in both the *apxIC* and *apxIIC* genes. The protoxins ApxIA and ApxIIA are formed and exported but are not acylated, and thus not activated, due to the absence of the ApxC proteins. The bacterial strain was cultured on brain heart infusion (BHI) broth (Oxoid Ltd, Basingstoke, Hampshire, England) or on BHI agar supplemented with 5 or 15 μg/mL nicotinamide adenine dinucleotide (NAD) (BHI-NAD), respectively, at 37°C in 5% CO_2_. *A. pleuropneumoniae* MBHPP147 was transformed by electroporation with pMC-Express, a plasmid for GFP expression [15], as described before [20]. Transformants were selected on BHI agar with chloramphenicol (1 μg/ml).

### Cell culture

The St. Jude porcine lung epithelial cell line (SJPL) (St. Jude Children’s Hospital, Memphis, TN, USA) [21,22] was grown in Dulbecco’s modified Eagle’s medium (DMEM) (Gibco, Burlington, ON, Canada) supplemented with 10% of fetal bovine serum (Gibco), 1% sodium pyruvate (100×, Gibco), 1% L-glutamine (200 mM, Gibco), 1,5% MEM nonessential amino acids (100×, Gibco), 1% penicillin/streptomycin (100×, Gibco), 1% fungizon (250 μg/ml, Gibco) and 0,1% gentamicin [21]. Cells were grown at 37°C in 5% CO_2_. Despite its name, the SJPL cell line was recently shown to be from monkey origin [22].

### Cytotoxicity detection assay

For the cytotoxicity detection assay, 10^5^ epithelial cells in complete DMEM without antibiotics were seeded into wells of 24-well tissue culture plates (Sarstedt, Numbrecht, Germany) and incubated O/N. Cells were infected with *A. pleuropneumoniae* strain MBHPP147. Bacteria from an overnight culture grown at an OD_600nm_ of 0.6 were diluted in complete DMEM cell culture medium without antibiotics and supplemented with NAD (5 μg/mL) to a concentration of 10^6^ CFU/ml. One ml of this suspension was added to each well at a multiplicity of infection (MOI) of 10:1, and the plates were incubated for up to 96 hours*.* The cellular cytotoxicity was determined using the lactate dehydrogenase (LDH)-measuring CytoTox 96 nonradioactive cytotoxicity assay (Promega, Madison, WI) as prescribed by the manufacturer. Non-infected cells were used as a negative control, while total lysis of cells by a treatment with the lysis solution represented the 100%-cytotoxicity positive control. Optical densities were measured at 490 nm with a microplate reader and percentage of cytotoxicity was calculated using the following formula: (OD_490_ treated well/OD_490_ positive lysis control well) × 100.

### Biofilm assay with an abiotic surface

A static microtiter plate biofilm assay was used as described by Tremblay et al. [10]. The wells of a sterile 96-well polystyrene microtiter plate (Costar® 3599, Corning, NY, USA) were filled in triplicate with a dilution (1/100) of an overnight bacterial culture. Following an incubation of 4 to 24 h at 37°C, the medium was removed by aspiration and the wells were then washed by immersion in water. The water was then removed by aspiration and the excess water was removed by inverting plates onto a paper towel. The wells were then filled with 100 μL of crystal violet (0.1%) and the plate was incubated for 2 min at room temperature. After removal of the crystal violet solution, the plate was washed and dried in 37°C for 30 min and 100 μL of ethanol (70%) were added to the wells. Absorbance was measured at 590 nm using a spectrophotometer (Powerwave, BioTek Instruments, Winooski, VT, USA).

### Biofilm assay with a biotic surface

Bacteria (500 μl) from an overnight culture were transferred in 5 ml of fresh BHI-NAD and were grown at 37°C with agitation (200 rpm) until an OD_600nm_ of 0.6. Bacteria were then diluted to a concentration of 2.5 × 10^6^ CFU/ml in complete DMEM cell culture medium without antibiotics and supplemented with NAD. The medium was removed from wells of 96 well-microtiter plates containing confluent monolayers of SJPL cells and 100 μL of the bacterial dilution in DMEM-NAD were added (MOI of 10:1). Plates were incubated at 37°C in 5% CO_2_ for 3, 6, 24, or 48 hr. To disperse the biofilms, 100 μL of a Dispersin B solution (0.4 μg/mL in Dubelcco’s phosphate-buffered saline [DPBS]; Kane Biotech Inc., Winnipeg, MB, Canada) was added to the well and the plate was incubated for 5 min at 37°C. After the desired incubation period, culture medium was removed and the wells washed 3× with PBS.

### Quantification of *A. pleuropneumoniae* attached to SJPL cells

Adherent bacteria were quantified as described previously [13]. Briefly, SJPL cells were seeded into 24-well tissue culture plates as described in the cytotoxicity assay. Bacteria were prepared as described above and 1 ml of the bacterial suspension was added to each well. The plates were incubated for 3, 6, 24 and 48 hours. When necessary, bacteria in biofilms were dispersed by adding 100 μL of a Dispersin B solution (4 μg/mL in DPBS) to the well and the plate was incubated for 5 min at 37°C. Nonadherent bacteria were removed by washing four times with DPBS. Cell with adherent bacteria were released from the wells by adding 100 μl of 1× trypsin-EDTA (Gibco) and resuspended in 900 μl DPBS buffer. The recovered suspension was serially diluted and these were plated on agar to determine the number of adherent bacteria.

### Confocal laser scanning microscopy

Biofilms were grown on a biotic or an abiotic surface as described above. SJPL cells were fixed in formaldehyde (4%) for 1 hr at room temperature and wells were washed 3 more times with PBS. The wells were filled with 100 μl of WGA–Oregon green 488 or WGA-AlexaFluor 633 (Invitrogen, Eugene, OR, USA) diluted 1/100 in PBS or/and Phalloidin-AlexaFluor 594 (Invitrogen) diluted 1/40 in PBS and the plate was incubated for 30 min at room temperature in the dark. The plate was then washed with water and filled with 100 μl PBS. The plate was observed with a confocal microscope (Olympus FV1000 IX81, Markham, ON, Canada). The fluorophores were excited and detected as prescribed by the manufacturers. The images were acquired using the Fluoview software (Olympus).

## Abbreviations

BHI: Brain heart infusion; DMEM: Dulbecco’s modified Eagle’s medium; DPBS: Dubelcco’s phosphate-buffered saline; MOI: Multiplicity of infection; NAD: Nicotinamide adenine dinucleotide; PGA: Poly-β-1,6-N-acetyl-D-glucosamine; PRRSV: Porcine reproductive and respiratory syndrome virus; SJPL: St. Jude porcine lung; WGA: Wheat germ agglutinin.

## Competing interests

RPAMS is employed by MSD-Animal Health and involved in vaccine development. The other authors declare that they have no competing interests.

## Authors’ contributions

YDNT and CL carried out the cell culture, cytotoxicity, CFU counts and biofilm formation assays, confocal microscopy experiments, and participated in the writing of the manuscript. RPAMS constructed and characterized the non-hemolytic mutant strain of *A. pleuropneumoniae* and participated in the writing of the manuscript. MJ conceived the study, realized its design, supervised the trainees and drafted the manuscript. All authors read and approved the final manuscript.
